# Do emotions mediate the relationship between eating pathology and autistic traits?

**DOI:** 10.1186/2050-2974-3-S1-O25

**Published:** 2015-11-23

**Authors:** Salma Mansour, Vanja Rozenbladt, Matthew Fuller-Tyszkiewicz, Chiara Paganini, Janet Treasure, Isabel Krug

**Affiliations:** 1 University of Melbourne, Australia; 2 Deakin University, Australia; 3 University of Tasmania, Australia; 4 King's College, UK

## Introduction

An overlap between Eating Disorders (EDs) and Autism Spectrum Disorder (ASD) has been proposed based on clinical and behavioural observations. No research to date has however assessed the potential mechanisms linking the two disorder sets.

## Objectives

To assess the extent of overlap in levels of eating pathology and ASD traits and to explore potential mechanisms linking the two disorder sets by assessing the mediating effects of negative attitudes towards emotions and emotion dysregulation.

## Methods

401 university students (82.8% females), filled in an online questionnaire including the following measures: the Eating Attitudes Test (EAT-26), the Autism Spectrum Quotient (AQ), the Empathy Quotient (EQ), the Systemising Quotient (SQ), the Attitudes towards Emotional Expression Scale (ATEE), and the Difficulties in Emotion Regulation Scale (DERS).

## Results

With, the exception of Empathy and Systemising, all other ASD traits were positively related to ED symptoms (p<0.05). Multiple mediation analyses indicated that the indirect effect of AQ on EAT-26 through DERS was significant, indicating full mediation. Conversely, the indirect effect via ATEE was insignificant (close to zero) and therefore ATEE does only partially mediate the relationship between AQ and EAT-26.

## Conclusions

The association between EDs and ASD that has been identified in clinical samples extends into non-clinical samples. The mediation analyses results indicate that AQ operates on EAT-26 via DERS as a full mediator.

